# The paradoxes of telehealth platforms: what did we learn from the use of telehealth platforms?

**DOI:** 10.3389/fdgth.2024.1346039

**Published:** 2024-02-13

**Authors:** Khayreddine Bouabida, Bertrand Lebouché, Marie-Pascale Pomey

**Affiliations:** ^1^ Centre de Recherche, Centre de Recherche du Centre Hospitalier de l’Université de Montréal (CRCHUM), Montreal, QC, Canada; ^2^ École de Santé Publique, Département de Gestion, D’évaluation et de Politique de Santé, Université de Montréal, Montreal, QC, Canada; ^3^ Department of Family Medicine, Faculty of Medicine & Health Sciences, McGill University, Montreal, QC, Canada; ^4^ Centre for Outcomes Research & Evaluation, Research Institute of the McGill University Health Centre, Montreal, QC, Canada; ^5^ Chronic Viral Illness Service, Department of Medicine, Division of Infectious Diseases, McGill University Health Centre, Montreal, QC, Canada; ^6^ Infectious Diseases and Immunity in Global Health Program, Research Institute of the McGill University Health Centre, Montreal, QC, Canada; ^7^ Centre de Recherche, Hospital Center of the University of Montreal (CHUM), Montreal, QC, Canada; ^8^ Centre de Recherche, Centre of Excellence on Partnership with Patients and the Public, Montreal, QC, Canada

**Keywords:** COVID-19, telehealth, patient remote monitoring platform, patients, healthcare professionals, utilization, evaluation, paradox

## Abstract

This article is an overview and reflection of the findings of an evaluative study conducted on a program called “Techno-Covid Partnership” (TCP) implemented in April 2020 at the Centre Hospitalier de l’Université de Montréal (CHUM) in Montreal, Canada. In the context of the COVID-19 pandemic, the CHUM decided in April 2020 to implement telehealth, virtual care, and telemonitoring platforms and technologies to maintain access to care and reduce the risks of contamination and spread of COVID-19 as well as to protect users of health services and health professionals. Three technological platforms for telehealth and remote care and monitoring have been developed, implemented, and evaluated in real-time within the framework of the TCP program. A cross-sectional study was carried out in which a questionnaire was used and administered to users of telehealth platforms including patients and healthcare professionals. The methods and results of the study have been published previously published. In the completion of the two articles published in this context, in this paper, we briefly recall the context of the study and the method performed. The main focus of the paper is on presenting a critical overview and reflection on the major findings of our evaluation of the use of telehealth platforms from the point of view of patients and health professionals and discuss certain paradoxes i.e., the advantages, challenges, recommendations, and other perspectives that emerged in this study.

## Context

**Figure 1:**
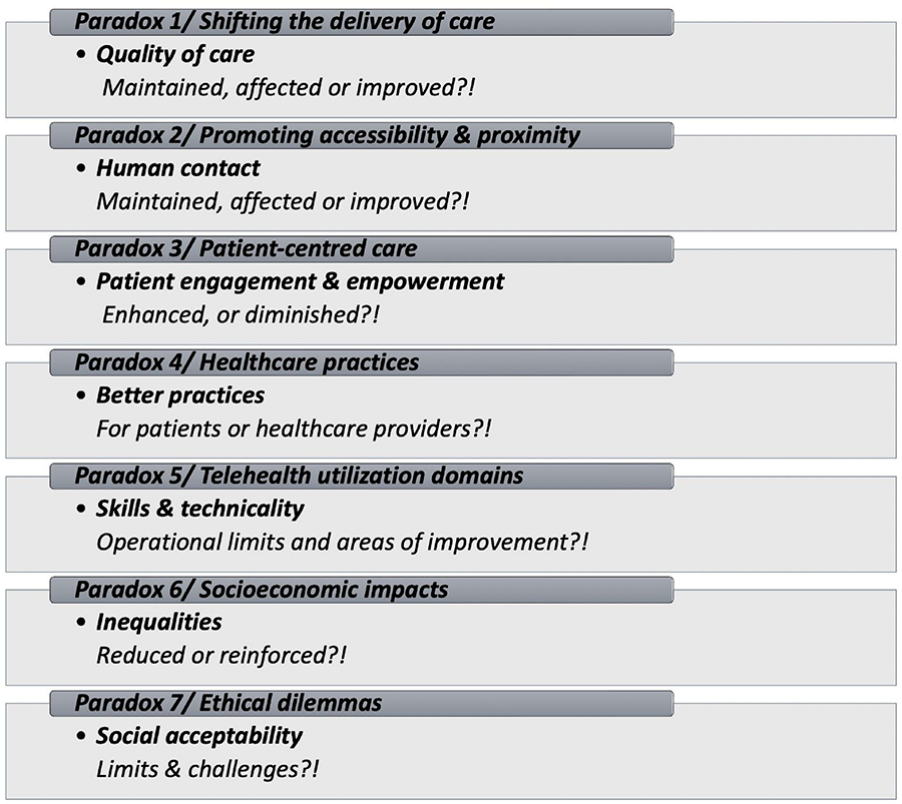
The telehealth platforms use’ paradoxes summary.

The COVID-19 pandemic has created an urgent need for action to reduce the spread of the virus and reduce congestion in health services, protect caregivers, and help them maintain satisfactory quality and safety of care. Telecare and telemonitoring platforms quickly emerged as potential solutions. Thus, at the University of Montreal Hospital Center, three platforms have been set up: (1) remote assistance (Telecare-Covid) provided by nurses and doctors by telephone calls, and (2) remote monitoring (CareSimple-Covid) which allows remote monitoring of the patient at home, and (3) a teleconsultation platform (React-Teleconsultation) to carry out visual consultations between professionals and patients. In this context, two cross-sectional studies were performed between July and September 2020. The first was carried out with patients who had used one of the two, Telecare-Covid and CareSimple-Covid platforms. Questionnaires were given to the patients by telephone. The data collected was analyzed using descriptive statistics and *t*-test analysis. The second focuses on the use by professionals of Telecare-Covid and React-Téléconsultation. The data was collected by email from healthcare professionals. The data were analyzed using SPSS software. Fisher analyses were performed to compare perceptions of performance, safety and quality, issues, and perceived problems among healthcare professionals. In this paper, and after completing the two articles previously published (1, 2), we directly present a critical reflection of certain dimensions that could be perceived as paradoxes that emerged from our study. Even though the study has been performed during the global pandemic context of COVID-19, the 7 paradoxes we present may still be worth consideration in a normal global context. All these paradoxes are legitimate and carry with them certain questions worth asking and some dilemmas that can be addressed (Figure 1).

## The 7 paradoxes

### Paradox 1: shifting the delivery of care without affecting the quality of care?

***“*** *Delivery of care vs. Quality of care: This paradox lies in how realistic shifting the delivery of care to a virtual setting is, pursuing maintaining or even improving the quality of care provided.”*

Although its use existed long before the beginning of the COVID-19 pandemic, it should be noted that the use of telehealth has greatly increased because of the pandemic. Many papers in the literature review all agree to claim that the global health emergency of the COVID-19 pandemic has led to significant changes in the management and delivery of care through the deployment of health measures, including physical distancing and stay-at-home orders (3, 4). This situation has forced health systems, including hospital managers and health professionals, to adapt their practices to respect barrier measures and strengthen the health safety net while ensuring continuity and optimal quality of care in very specific circumstances (3, 4). Telehealth has been the modality for organizing and delivering basic care services. It is complementary to conventional modalities that health systems have used the most to strengthen their strategy of change and adaptation in the context of the COVID-19 pandemic [4.5]. Telehealth has made it possible to reduce certain inequalities by making services traditionally offered in person accessible (5–8). Using secure digital tools, in particular mobile devices (tablet, smartphone, computer, etc.) connected to systems in the form of specialized technology platforms designed for careful monitoring, examinations, and remote patient follow-up, it was possible for both the healthcare professionals to provide their care and for the users to receive it in safer settings (5–8). Overall, the delivery of care without affecting the quality of care has been changed to consider the pandemic context. However, some questions remain unanswered and deserve further consideration (5–8). For instance, how has this strategically and operationally happened, and what exactly has changed what? Were there any other variables that have influenced or contributed to that shift other than the pandemic conditions, such as safety, restrictions, measures, etc.? Were there other players or circumstances helping to shift that dynamic that we missed identifying?

### Paradox 2: promoting accessibility and proximity at the expense of human contact?

***“*** *Accessibility VS Human Ccontact: This paradox lies in its ability to promote accessibility and proximity to healthcare services while potentially reducing the level of human contact inherent in traditional face-to-face interactions.”*

Among the advantages and strengths most appreciated by users on all the telehealth platforms we evaluated and among most health professional users and patients alike is the ease of access to care. But also, an interesting point raised by many participants is that in the context of a health emergency, the ease of accessibility at any time to care by these platforms is considered as proximity. This means that in such a pandemic context filled with uncertainty, fear, and physical and psychological barriers, it is still possible always to offer accessible care, and continuously offering a mix of synchronous and asynchronous care would be relevant. Asynchronous care makes easy access and brings it close to patients. In fact, patients believe that they are “close” to health professionals despite the distance and the circumstances. Similarly, healthcare professionals say they feel “close” to their patients, which has created a feeling of assurance and trust between patients and healthcare professionals on telehealth platforms. Proximity, which differs from accessibility, is one of the essential dimensions we have raised in our study, but also in a few other studies where we have defined what is classically understood by proximity. The latter is defined as when you have easy, guided, engaging, and integrated access to care. Above all, continuous and without interruption and with various clinical and medical disciplines (1, 2, 5–10). The proximity of care can be physical or virtual, which is the case for the platforms we studied (1, 2, 5–10). Some writings consider that proximity in telehealth refers to the ease and continuity of access to care but also to secure, continuous, and real-time communication and exchange of information between patients and caregivers in very specific circumstances such as those of the COVID-19 health emergency mediated using technological platforms (1, 2, 5–10). Still in the dimension of accessibility, but on a technical aspect, our study has shown that users give much importance to user-friendliness and technical ease of use. Patient and healthcare professional users obviously prefer dynamic, simple, user-friendly platforms compatible with the care provided and received. In some studies of the literature, it is considered that user-friendliness and ease of use rank first as preferences even before the multitude of services offered and data security as well as other technical characteristics and features of telehealth use (1, 2, 11, 12).

### Paradox 3: have telehealth platforms enhanced or diminished patient-centred care?

***“*** *This paradox lies in the potential to diminish or enhance patient-centred care by improving convenience, accessibility, and patient engagement, knowing the challenges related to the telehealth technology divide, communication quality, and technological barriers.”*

In contemporary care delivery, management, and organization models, there is a strong focus on patient engagement to continuously improve the quality of care. Certain quality constituents of care, such as accessibility, proximity, safety, equity, and efficiency, greatly facilitate patient engagement in care (13, 14). The results are encouraging in this dimension, and all the telehealth platforms we studied showed some degree of patient engagement. The elements evaluated in the quality dimension demonstrate remarkable appreciation by patients and health professionals (up to 85%). The results of our study suggest that these platforms have made it possible to maintain a satisfactory level of quality and safety in the continuum of care. Most users of the telehealth platforms studied, particularly health professionals, affirmed that they felt safe and protected against contamination by COVID-19 thanks to the use of telehealth platforms. As for the performance of the health professionals on the platforms, most consider that their performance has not been affected much apart from some minor technical problems, such as short network cuts or connection interruptions and platform freezes, which often end up resolved very quickly. Some professionals have indicated that even if some encounter technical difficulties, they still prefer to use these platforms rather than continue and provide their care to patients in person, with the risks of transmission that this can represent for the patient and care providers. Patients have generally assessed positively the quality and safety of the care and follow-up offered on the telehealth platforms. Most patients did not experience a sharp drop in performance or observed flaws or even negligence in the performance of professionals. On the contrary, most patients claimed to have received care according to the standards to which they are accustomed and sometimes even better, thanks to the ease of access. In addition, patients also claim to be well integrated and committed to their care by caregivers and, therefore, satisfied with the care received. Nevertheless, a minority of patients highlighted an important element of the quality dimension they felt was sometimes missing when using and being followed through the telehealth platforms. This concerns the human aspect when interacting with healthcare professionals. However, these patients say they don't know if this is naturally due to the virtual care process, which can feel cold and emotionally superficial due to the virtual distance, or if it's because of the drop in motivation and the desire to demonstrate more emotions and empathy virtually on the part of professionals towards patients. We were unable to verify this in depth. Is it directly related to the performance of healthcare professionals toward patients? Is this a normal effect associated with the virtual nature of the interaction between healthcare professionals and patients? Do professionals express less emotion and empathy? Is this simply a wrong perception and or misinterpretation by patients? Some studies find that some can perceive virtual human social interactions as less interesting than in-person human interactions in all areas (1, 2, 11, 12, 15–17). As a result, we can assume that this is most likely due to the very nature of virtual interaction, which can sometimes diminish the human aspect that man can perceive, feel, and develop toward others during an interaction at a virtual and or distance in a socio-professional context (15–19).. Furthermore, we could also reflect and ask questions on how this has affected the patient's self-management, particularly for chronic conditions. The importance of self-management has been further highlighted by the COVID-19 pandemic, which introduced additional barriers to accessing regular follow-up (18). More questions in this matter how the patient has been engaged and how the patient could have been better engaged. can be further explored.

### Paradox 4: better practices, for who? patients or healthcare providers?

***“*** *Healthcare Provider vs. Patient: This paradox lies in the extent of the change brought by telehealth use on both healthcare providers and patients and who benefits the most.”*

The study of users' perspectives has also shown us that beyond improving accessibility and the quality and safety of care, which have indeed been directly perceived and identified impacts, these platforms appear to have offered indirect effects that also have other benefits and better practices for patients and healthcare professionals. The participants in our study indicated that using these platforms, despite some limitations, gave them more autonomy, freedom, and tranquillity in using care services and better conditions for communication, collaboration, and teamwork. This was mainly felt among young health professionals but also among young patients. Indeed, the users of these platforms claim to have regained a certain autonomy that they did not find in the traditional use of care services. Patients have claimed that these platforms can organize their care program and episodes with the professional dynamically and smoothly, without going through a whole administrative and logistical phase. This allowed some patients to avoid commuting and transportation to clinical settings. For others, this has saved them long waiting hours and reduced their frustration when using the classical in-person care services due to the waiting time and administrative procedures they may go through before even meeting the healthcare professional. This could then reflect on their state of mind, which may reduce their engagement and affect their ability to communicate and collaborate appropriately once in the healthcare professional's office.

Similarly, among health professionals, using these platforms has given them more autonomy and control over their professional practice. The flexibility and dynamism of telehealth platforms have facilitated their work and reduced their frustration by allowing them to collaborate better and communicate with patients and their colleagues. Some health providers claim that the telehealth platforms offer them tranquillity, confidence, and openness but also a saving time, allowing communication to be more effective, clear, and open between them and their patients. These platforms, whether directly or indirectly, may or may not effectively offer better practices in the delivery of care for patients also for healthcare providers, which suggests further studies and evaluation as this has been recommended and encouraged by many experts in this field (1, 2, 10–20).

### Paradox 5: technical and operational limits: telehealth utilization as a skilled domain?

***“*** *Easy Utilisation VS Skilled Domain: This paradox lies in the fact that telehealth has been initially introduced for its friendliness and easy use but also requires some technical skills and technology literacy.”*

Several technical issues have been identified in our study, which are essentially limited to technical features and operational flaws specific to the functionalities of the platforms (e.g., the scope of the services offered, the connection, the applications or platforms of the function, speed of responsiveness, bugs, and interruptions, access to connected digital devices, etc.) that the engineers, and managers of the platforms have promptly taken into consideration and corrective measures have been implemented. Some users encountered other issues (e.g., entering clinical and medical data, viewing notifications from healthcare professionals, and managing follow-up alerts, etc.), especially on first use. These users claimed that these difficulties disappeared with the support and guidance of the technical staff of the telehealth platforms. It may seem odd or exaggerated, but we learned from the users that proper and smooth utilization of telehealth platforms requires some skills. The lack of training and support was highlighted by many participants, particularly among healthcare providers. You should know that healthcare providers are often skeptical of virtual care and data security (ref). With better training and support from management, these professionals could have a more favourable position on these issues. Moreover, some studies suggest that training in the use of digital health platforms, whether among patients or health professionals, is one of the main determinants of acceptability and adherence to the use of virtual platforms for care and remote monitoring (1, 2, 17, 18). These studies have found that training is an organizational issue and that users who are well-trained in using digital platforms feel well-equipped, confident, and comfortable and express less rejection, mistrust, and anxiety in using telehealth platforms (1, 2, 17–19, 21–23).

### Paradox 6: telehealth, to reduce or to reinforce socioeconomic inequalities and beyond?

***“*** *This paradox lies in the impact of telehealth on socioeconomic inequalities, which is multifaceted and can either reduce or reinforce existing disparities, depending on various factors. Here's a nuanced perspective.”*

Our study certainly covered only some of the dilemmas and issues in this area. However, we have thought about the issues that were not raised directly by the participants, but which remain conceivable from our point of view. For example, from a public health perspective, it can be assumed that some observers could judge the use of telehealth platforms via screens and connected devices as a means that encourages a sedentary lifestyle (18, 22–29). Knowing that sedentarily is an important factor associated with many health problems, including obesity, which increases the risk of cardiovascular disease (19, 21–29). Using these platforms could promote isolation and weaken interactions and social bonds, reducing cohesion and bringing individuals together, particularly in clinical settings. We can also think about economic and environmental issues which can be indirectly linked to the meteoric rise in the use of telehealth platforms. Travel and transportation are indeed reduced with the use of telehealth platforms, but the use of these platforms also requires sophisticated technological systems and materials manufactured in industries, without forgetting, of course, that the use of these platforms requires maintenance and physical equipment and energy, which cannot be without effect on the environment (22–30). In terms of the economy, the large-scale use of these platforms could, in the long term, eliminate jobs, particularly in infrastructure support and maintenance, administration, transport, etc. Moreover, there is already a manpower shortage in the medical profession, especially among doctors and nurses. These platforms can free caregivers from repetitive tasks if patients which to receive the information themselves through these platforms and rather concentrate on other tasks where they are more useful. So remote care and monitoring platforms are certainly a genius invention with a lot of potential and advantages, but the public health perspective forces us to take human advances in all areas with caution. We encourage the proper use of these technological platforms, and we think about all the possible long-term issues to find strategies to regulate them as best as possible to prevent and ensure better use. Ultimately, the goal is to maximize the positive effects for which these platforms were originally developed. Ultimately, these issues should not demotivate or disinterest us in continuing to develop and promote the use of telehealth platforms. On the contrary, we should seriously consider these issues and increase our efforts to ensure their continuous improvement meets the needs and expectations of users and support healthcare systems.

### Paradox 7: what about ethical challenges and social acceptability limits?

***“*** *This paradox lies in the numerous ethical challenges and social acceptability limits revolving around balancing the potential benefits of telehealth with the ethical considerations and societal norms that shape its implementation.”*

The most important issues and areas for improvement that emerged in our study are not of a technical or operational nature but rather of a social, organizational, and public health nature. Indeed, issues related to social acceptability in maintaining human contact in care, data security, and user training and support deserve great attention and should be addressed thoroughly and with caution. While acknowledging the potential and benefits the platforms have demonstrated, some participants, whether patients or even healthcare professionals, still insisted on maintaining human contact when providing care. These participants consider that nothing can replace human contact, especially when one is suffering and in a fragile physical, psychological, and emotional situation. For them, direct, in-person human contact can make much difference compared to virtual contact. Furthermore, for other participants, although the elaborate regulatory system approved by the CHUM institution and its research center CRCHUM regarding maintaining confidentiality and user data security, the issue of confidentiality and data leaks was nevertheless present and remained a concern. This minority of participants, even if they understand the health emergency, still prefer in-person care delivery to better secure their data and for more confidentiality. Moreover, even knowing that the CHUM and the development, management, and maintenance teams of the platforms have fully complied with data security and confidentiality measures and that no incident of this kind has been reported or observed, this minority still prefers in-person care delivery. However, some people may still express different concerns and views on this issue, which is socially understandable. A final point that participants sometimes raised, particularly patients, and even though they trust that their data is safe and protected, they still consider that they feel uncomfortable that a non-medical staff, in particular the technical and operational support team, can consult their data and information during interactions and communications to resolve technical problems if ever happens. So, once again an important issue that must be addressed with an ethical approach and a multidisciplinary perspective.

## Discussion

The telehealth platforms studied have not been the subject of an implementation evaluation to identify in depth the factors limiting or favouring their implementation. However, through our experience in this environment during all the phases of the deployment and evaluation of these platforms, and thanks to the numerous writings we consulted, we could briefly discuss certain dimensions related to this question. Following our experience acquired through our study, we would like to shed some light on the most important factors that promote or limit the implementation of telehealth in general. We have demonstrated in this study that telehealth is interconnected between several sectors.

### Culture

To promote telehealth, consideration should be given to establishing mechanisms for raising awareness, dialogue and exchanging expertise and information between the stakeholders and actors involved. The classic professional culture and resistance to change, not to mention the lack of organizational capacity, the will and support of decision-makers, the lack of vision to innovate, and of course, the lack of training and support for healthcare teams and patients to use them, are among the main factors that limit the implementation and maintenance of the long-term use of telehealth platforms. One approach that can help reduce resistance to change and break down barriers between different stakeholder groups is implementing mechanisms for meaningful dialogue about potential innovations and reforms. Training, recognition, and financial incentives can be effective strategies that greatly facilitate and maintain the implementation and use of telehealth in health services. Improving user-friendliness and technical ease of use, promoting access to technology, and ensuring its continuous improvement and modernization greatly promote telehealth platform implementation, adherence, and long-term maintenance (17–19, 30–33).

### Training

Training is an essential determining element that promotes the adoption and adherence to the use of telehealth platforms. It is therefore strongly recommended to support and train healthcare professionals and patients before their first use. Patients and healthcare users must be systematically informed about the security and confidentiality of their personal data. In addition, institutions should better explain and communicate their regulatory standards and ethical principles to the public in order to reassure them and reduce rejection and skepticism regarding the use of telehealth platforms. Regarding maintaining human contact when providing care, we recommend engaging in discussions and consultations with patients, healthcare professionals, the public and experts in public health, ethics, technology, and politics to address this issue in a transparent and democratic deliberative process. In addition, integrating the 4P participatory approach (Precise, Predictive, Personalized, Preventive) during the development and implementation of telehealth platforms would be a fundamental asset. The 4Ps approach would better help healthcare providers and other interested parties make the most informed decisions while providing users with greater understanding and control over their choices about how to be monitored and receive care, whether remotely, virtually or in person (1, 2, 18, 24, 27–31). Finally, research in this area should be encouraged, and studies that focus not only on the questions raised in our study should be facilitated and supported. We should also have a broader look that considers the medium and long-term impacts on the healthcare system and public health in general (18, 30–33).

### Ethics

We emphasize that particular attention must be paid to ethics. The ethical factor is a major determinant that could alone limit or favour and promote the implementation and use of telehealth platforms in the short and long term. We have already stressed that it is very important to be attentive to the ethical issues that may arise from the use of telehealth. If ethical issues are not addressed in a clear, comprehensive, and satisfactory manner, the willingness of the public and professionals to use these technologies is likely to be reduced. For example, we expect guidelines to frame professional practice in tele-practice will be determined. As well as clear ethical guidelines are developed on issues such as equal access to telehealth technologies and confidentiality regarding access to medical records by non-medical personnel, for example, in call centers by engineers and technical support staff. It is also important to define and specify the knowledge and skills required to ensure that the services provided to patients through telehealth platforms meet the highest quality standards. There are also ethical issues around transparency, informed consent, dignity, open information, and oversight of data management through these virtual platforms. It is also important to determine the standards for managing the digital file, particularly on the confidentiality and integrity of data related to the use of telehealth platforms (30–33). It would be necessary to determine as best as possible the effectiveness of the benefits and the risks associated with using telehealth platforms and to communicate them clearly to users to help them make their choices. Finally, the big question that ethics must address concerns the maintenance of human contact and the humanization or virtualization of care on telehealth platforms to strengthen adherence to the use of telehealth.

## Conclusion

A crisis might be seen as a good window of opportunities, and let's say COVID-19 has created this window to promote and innovate in the telehealth domain (30–33). Telehealth platforms have offered on one hand numerous advantages that we should reinforce and explore further and on the other hand raised issues that need to be seriously considered and addressed in depth if we want to continue to use them on a large scale and achieve better results. Better results, clinically in terms of health outcomes but also strategically i.e., better organization and allocation of healthcare structures and resources and of course socio-ethically with better social acceptability of telehealth use. Progress is still being made, particularly on ethical and social acceptability issues but also in terms of accessibility to telehealth technology. For instance, efforts to ensure patient data privacy and security have intensified. Regulations like HIPAA (Health Insurance Portability and Accountability Act) in the United States and GDPR (General Data Protection Regulation) in Europe mandate strict guidelines for handling health data. Telehealth platforms are increasingly implementing robust encryption and authentication protocols to safeguard sensitive information. Furthermore, to improve inclusivity and accessibility, initiatives are underway to make telehealth services accessible to a broader population, including those with disabilities, limited internet access, or language barriers. Regarding the progress on ethical issues, medical associations, and healthcare organizations are developing ethical guidelines and professional standards specific to telehealth practice. These guidelines cover issues such as patient consent, informed decision-making, maintaining confidentiality in virtual consultations, and ensuring the quality of care delivered remotely. Moreover, efforts to raise public awareness and educate both patients and healthcare providers about telehealth benefits, limitations, and best practices are ongoing. This includes disseminating information through online resources, training programs, and public health campaigns to promote informed decision-making and responsible use of telehealth services.

To achieve more improvement, in the telehealth and digital health domain, the seven paradoxes that emerged from our study, and their areas of improvement should be considered. Taking these paradoxes into account could help us prepare for a probable new pandemic in the future. We encourage a growing body of research exploring the efficacy, safety, and patient outcomes associated with telehealth and digital health platforms and interventions. Evidence-based practice guidelines should be developed to inform healthcare providers regarding the best practices of telehealth use across various medical specialties and domains. Considering the elements explored in this paper and as a final perspective, we hope to encourage the reader's reflection and encourage exchange and collaboration between specialists, patients, citizens and experts, and all stakeholders interested in the field of telehealth. Overall, true progress in the telehealth and digital health domain will be attained through collective and collaborative efforts involving stakeholders from the healthcare industry, regulatory bodies, technology companies, and advocacy groups to ensure that these innovations serve the best interests of patients while upholding ethical principles and societal values.

## Data Availability

The raw data supporting the conclusions of this article will be made available by the authors, without undue reservation.
